# A Method to Determine the Habit Plane of a Dislocation Loop

**DOI:** 10.3390/ma19030497

**Published:** 2026-01-26

**Authors:** Yufeng Du, Lijuan Cui, Xunxiang Hu, Farong Wan

**Affiliations:** 1 National Key Laboratory of Nuclear Reactor Technology, Nuclear Power Institute of China, Chengdu 610213, China; bkxiaodu107@163.com; 2 School of Materials Science and Engineering, University of Science and Technology Beijing, Beijing 100083, China; wanfr@mater.ustb.edu.cn; 3 College of Physics, Sichuan University, Chengdu 610064, China; huxx@scu.edu.cn

**Keywords:** nature of dislocation loops, habit plane, inside-outside method, TEM

## Abstract

The nature of dislocation loops significantly influences their evolutionary behavior and, consequently, affects the material properties, particularly under irradiation conditions. Determining the habit plane of a dislocation loop is the key point to examining its nature using the inside–outside method. In the present study, we introduce a novel technique for determining the habit planes of dislocation loops in the transmission electron microscope (TEM). The traditional inside–outside technique requires an edge-on perspective of the dislocation loop for analysis of the habit plane. In contrast, our innovative method for the precise determination of the habit plane delves into the geometric correlations between the dislocation loop and its projections under different crystal zone axes in TEM without being bound by the restrictive requirement of an edge-on view. It also simplifies the procedure of the inside–outside method. Furthermore, we have discussed the advantages and limitations of various methodologies employed to examine the nature of dislocation loops, as well as the techniques for determining their habit planes.

## 1. Introduction

According to the design of fusion reactors and Generation IV fission reactors, the structural materials and the core components require excellent performance under intense neutron irradiation at high temperatures [1,2]. The dislocation loop is one of the most common irradiation-induced microstructures, which stems from point defects assembling [3]. During irradiation, the evolution of dislocation loops includes growing, shrinking, coalescence, recombination, and eventually some of them form dislocation lines or dislocation walls. Dislocation loops play a key role in material property degradation under irradiation. They significantly contribute to hardening and reduce the ductility of materials [4,5]. On the other hand, the recombination of interstitial dislocation loops with vacancy loops can reduce the defect density, as well as the swelling rate [3]. In addition, they can also affect the crack propagation [6] and interact with grain boundaries [7]. Therefore, to get a better understanding of the irradiation resistance of materials, it is essential to investigate the properties of dislocation loops, especially their Burgers vector and nature type, either being interstitial type or vacancy type. For engineering nuclear materials, controlling the dislocation loop population is a common method to achieve the desired balance of mechanical properties [8,9].

Determining the nature of a dislocation loop is always challenging work. Several methods have been proposed to examine the nature of a loop, including high-resolution TEM observation of an edge-on loop [10], simulation of loop contrast [11], high-voltage TEM (HVTEM) [12,13] observation, and the inside–outside method. The most direct way is the high-resolution TEM observation of an edge-on loop, which can observe an additional layer of atoms, i.e., an interstitial loop or a layer missing of atoms, i.e., a vacancy loop. However, the sample preparation is extremely difficult. The sample thickness needs to be thin enough that, under a high-resolution TEM mode, the atom arrangement can be seen clearly. Moreover, in such a thin film, the dislocation loop needs to be in an orientation that lies in an edge-on position in the TEM. Another limitation of using thin foils for TEM is the surface effect, which could modify the dislocation microstructure (dislocation loops escaping at the foil surface, dislocation lines straightening up normally to the foil). Because of these high demands for the TEM samples, the high-resolution observation method is not commonly used and a limited number of loops can be analyzed. The HVTEM observation method is performed through introducing point defects in the sample at certain temperatures and observing the evolution of loops [12,13]. At these temperatures, the electron irradiation-induced interstitial point defects are mobile enough and can migrate to pre-existing dislocation loops, while the counterpart of the vacancy point defects is sessile. Thus, under the electron irradiation in HVTEM, the vacancy loops would shrink their size and even disappear, while the interstitial loops would grow [12,13]. The limitation of this method is the availability of HVTEM, since currently there are limited high-voltage TEM in the world; as the author knows, four are located in Japan, two in South Korea, one in the US, and one in Europe. Also, the temperature range needs to be determined before the HVTEM observation. In addition, this experiment is irreversible for the sample. For the simulation of the dislocation loop contrast method, it is commonly used when analyzing small dislocation loops within 5 nm [11]. The small dislocation loops are black dots of certain shapes with shadows in the TEM bright field image. The shape of the black dots depends on the reaction of the electron beam with the dislocation loop and the matrix in the surroundings. Through the simulation of this reaction, the figure of different types of dislocation loops can be obtained. By comparing with the loop contrast in TEM, the nature of the dislocation loop is revealed. This method is indirect, and additional simulation work is needed. In general, it is only used to analyze the small dislocation loops that cannot be distinguished by the inside–outside method. For bigger loops, the inside–outside method is suitable to analyze the nature [11,14,15,16,17,18]. To accurately determine the nature of a dislocation loop by the inside–outside method, in principle, it is necessary to know the Burgers vector and habit plane of the dislocation loop. Contrast theory shows that dislocation loops with different Burgers vectors will exhibit no contrast or little contrast when imaged under certain two-beam diffracting conditions of ***g***·***b*** = 0, where ***g*** is the operational diffraction vector and ***b*** is the Burgers vector of the dislocation loop [11]. However, the habit plane of a dislocation loop is difficult to determine. An edge-on contrast can tell the approximate habit plane of a loop. However, the title angle of the TEM holder is limited; on the other hand, even though the loop could be tilted to an edge-on position, this means that it is easy to flip over and the normal direction of the habit plane would change to the opposite direction according to the finish–start/right-hand (FS/RH) convention. In this case, it is easy to obtain the wrong nature type.

In the present study, a new and precise method is introduced to determine the habit planes of dislocation loops without requiring an edge-on view in TEM. The habit plane is determined through the geometric relationship between dislocation loops and matrix crystal orientations. The longest axis direction of the projected loops in different zone axes is used to examine the habit plane. This method simplified the procedure of the inside–outside method for determining the nature of dislocation loops. In addition, the application of the inside–outside method in different cases is discussed in detail.

## 2. Experimental Procedure

A model alloy of Fe-10 at. % Cr alloy is used to study the dislocation loops, which were made by arc melting and followed by heat treatment at 700 °C for 1 h. Disk samples with 3 mm in diameter were fabricated by mechanical polishing. The final thin foil samples for TEM analyses were prepared by twin-jet electro-polishing in Tenupol-5 at −20 °C using an electrolyte of 5% HClO_4_ and 95% ethanol (volume) solution [19]. Hydrogen implantation with the energy of 30 keV was performed on the TEM foil samples at room temperature to 1 × 10^17^ ions/cm^2^.

## 3. Results

### 3.1. The Projection of Dislocation Loops in TEM

**Figure 1:**
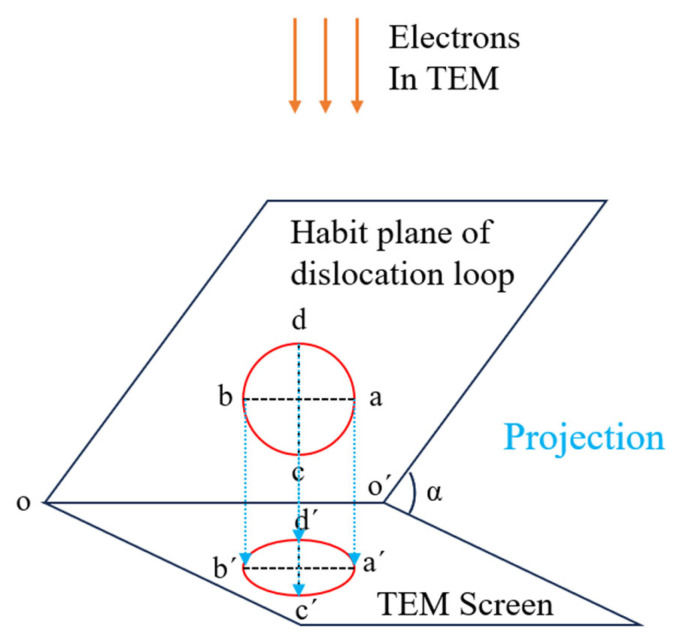
The projection of a dislocation loop in TEM.

Figure 1 shows the schematic map of the projection of a round loop on the view screen in TEM. When the dislocation loop is vertical to the screen, the image of the loop can be either in a line shape or a double-bean shape. When the habit plane of a loop is parallel to the screen, it keeps the original shape. In the common case, without considering the inside–outside contrast, the projection of dislocation loops in TEM is simple. According to the geometry projection relationships between the dislocation loop and its projected image, as shown in Figure 1**,** the longest axis of the loop projection is parallel to the intersection line between the habit plane of the loop and the screen, specifically a′b′//oo′, while the shortest axis is perpendicular to the intersection line, specifically c′d′⊥oo′. Meanwhile, the lengths of a′b′ and c′d′ can be calculated; a′b′ = ab and c′d′ = cdcosθ.

**Figure 2:**
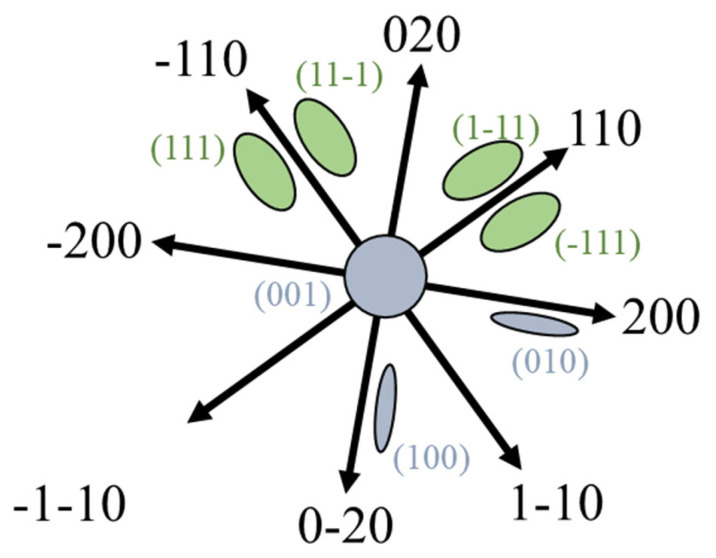
The projection of ½<111> {111} and <001> {001} loops under [001] zone axis in bcc structural materials. The green circles denote dislocation loops lying on {111} planes, while the grey circles represent those on {001} planes. The arrows represent the g vectors.

As an example, Figure 2 shows the sketch of pure edge ½<111> {111} and <001> {001} loops under [001] zone axes in bcc structural materials. Researchers always use these projected figures to determine the Burgers vector of dislocation loops in bcc Fe-based materials. This method assumes that only ½<111> {111} and <001> {001} loops exist in the material. The Burgers vector of dislocation loops in bcc Fe-based materials has already been determined to be either ½<111> or <001> from both experimental studies and simulations [20,21,22,23,24]. The habit plane of <001> loops also reach agreement to be {001} of pure edge type, while there are some arguments in the habit plane of ½<111> loops. Some simulation works showed that both {111} and {011} habit planes can be possible for ½<111> loops [25]. However, this simulation result has not been confidently approved by experimental studies, even though some TEM work shows that the edge-on image indicates their habit plane to be {112} or {011} [26]. This is because the edge-on contrast is not always reliable, which will be discussed in Section 4.

### 3.2. The Method to Determine the Habit Planes of Dislocation Loops in Bcc Materials

To provide a better illustration of the method for determining the habit plane of a dislocation loop, here we take a random dislocation loop in the irradiated model alloy of Fe-10 at.% Cr alloy as an example and illustrate the method step by step.

In this study, we adopt the right-hand rule to define the positive sense of the dislocation loop and the crystal coordinate, and employ the FS/RH (finish–start right-hand) convention for defining Burgers vectors. The zone axis and the habit plane of loops are designated as upwards.

#### 3.2.1. Labeling the Diffraction Vectors in Reciprocal Space

In practice, the crystal orientation is labeled according to the diffraction patterns. Therefore, correctly indexing the diffraction patterns in the reciprocal space is the fundamental issue for analyzing the nature of dislocation loops. However, in TEM the diffraction patterns sometimes exhibit a 180° rotation compared to real space, which is caused by adopting an odd number of lenses following the objective lens in the microscope column. For an even number of lenses, the real space and the reciprocal space do not have an inverse relation. The present TEM JEOL 2010 used for the characterization of dislocation loops does exhibit an inverse relation between the image and diffraction mode, and corrections in the diffraction maps were made.

**Figure 3:**
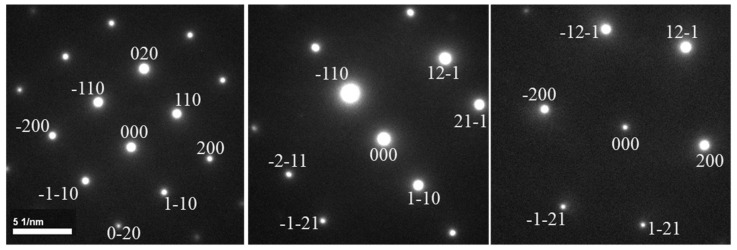
The diffraction patterns in three different zone axes in bcc structural materials.

**Figure 4:**
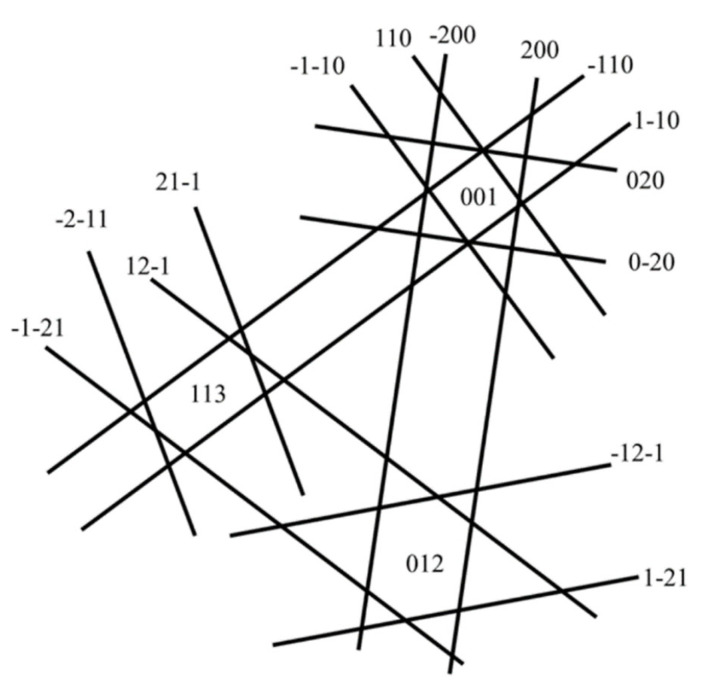
The Kikuchi pattern involving the three diffraction zone axes.

Since the zone axes in bcc materials are not always symmetrical, at least three nonlinear correlation zone axes are needed to index the diffraction patterns. The diffraction vectors are labeled in Figure 3, according to the Kikuchi maps in Figure 4.

#### 3.2.2. Examining the Burgers Vector of a Dislocation Loop

**Figure 5:**
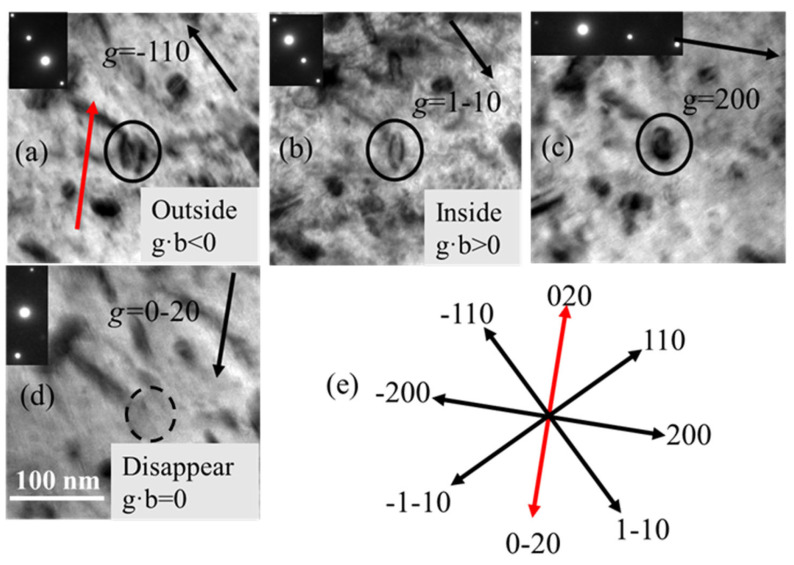
The TEM images of a dislocation loop under [001] zone axis with two-beam diffraction vectors of (**a**) ***g*** = −110, (**b**) ***g*** = 1–10, (**c**) ***g*** = 200, (**d**) ***g*** = 0–20, (**e**) vectors in [001] zone axis.

Numerous studies have reported that in bcc Fe-based alloys, only two types of dislocation loops formed, possessing Burgers vectors of either ½<111> or <100> [13,19,22,23,27]. Taking advantage of this conclusion, and combining the ***g***·***b*** invisible criterion ($s_{g}>0)$, we can determine the Burgers vector of the dislocation loop marked in Figure 5. Since the loop in Figure 5 is visible under diffraction ***g*** = −110, ***g*** = 1–10, and ***g*** = 200 and invisible under ***g*** = 0–20, through simple ***g***·***b*** calculations, we obtain that the Burgers vector of the dislocation loop is ±[100]. Moreover, when ***g*** = −110, the marked loop shows outside contrast in Figure 5a, specifically ***g***·***b*** < 0, and when ***g*** = 1–10, the marked loop shows inside contrast in Figure 5b, namely ***g***·***b*** > 0, so the Burgers vector of the marked loop is examined to be [100].

#### 3.2.3. Determining the Habit Plane of the Dislocation Loop

Under the [001] zone axis, ±[100] (100) pure edge dislocation loops should exhibit an edge-on view, appearing as either a double-bean or line-shape contrast. But, under the [001] zone axis, the projected shape of the analyzed dislocation loop in Figure 5 is elliptical, indicating that it is not a pure edge loop. However, within the tilting limitation of a normal double-tilt TEM holder, no edge-on view of this loop has been observed. To determine the habit plane of such a dislocation loop, a new method is proposed and demonstrated in the following.

**Figure 6:**
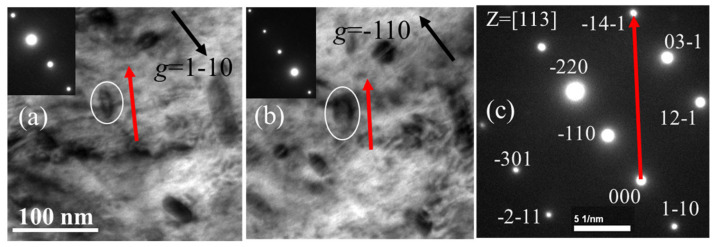
The TEM images of the dislocation loop under [113] zone axis with two-beam diffraction vectors of (**a**) ***g*** = 1–10, (**b**) ***g*** = −110, (**c**) diffraction pattern and −14–1 vectors in [113] zone axis.

**Figure 7:**
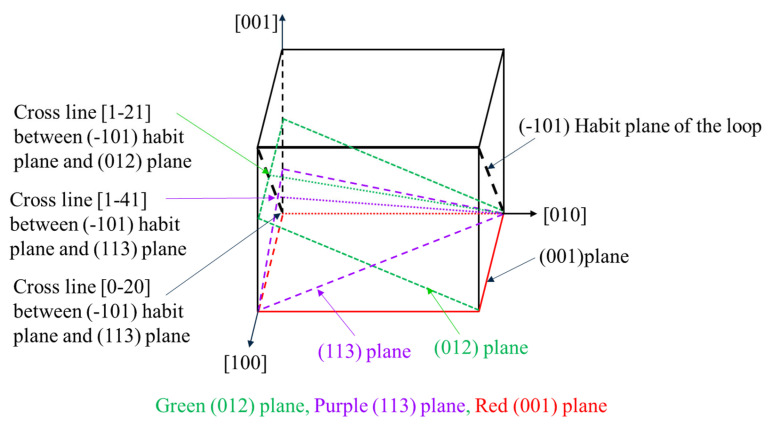
A schematic of dislocation habit plane (−101), projected screen of (001), (012), (113), and the cross lines between them.

As indicated by the red arrow in Figure 5a, under the [001] zone axis, the longest axis direction of the dislocation loop is in the [020] direction, which corresponds to a’b’ in Figure 1. Since a’b’//oo’// ab, [020] is also the intersection line direction of oo’ between the habit plane and the (001) view plane, as well as the line direction of ab within the habit plane. From the geometry relationship, we know that two nonlinear lines can determine a plane. Therefore, if two line directions can be obtained, the habit plane of the dislocation loops can be calculated. Then, the sample was tilted to another zone axis [113], as shown in Figure 6. A pair of two-beam condition images was acquired with ***g*** = ±1–10. From the shape of the dislocation loop, it tells that the intersection line between the habit plane and (113) plane is along the [1–41] direction; in the same way, [1–41] is also a line in the habit plane. Thus, until now two nonlinear lines in the habit plane have been obtained. The normal direction of the habit plane should be ***n*** = ±[0–20] × [1–41] = ±[−202] = ±[−101]. Here, we define the habit plane direction as the one at an acute angle with the vertical direction, namely the opposite electron beam direction. Therefore, the habit plane of the dislocation loop is (−101). Figure 7 shows the geometry relationship between the dislocation loop with the (−101) habit plane and the projected planes of (001) and (113).

**Figure 8:**
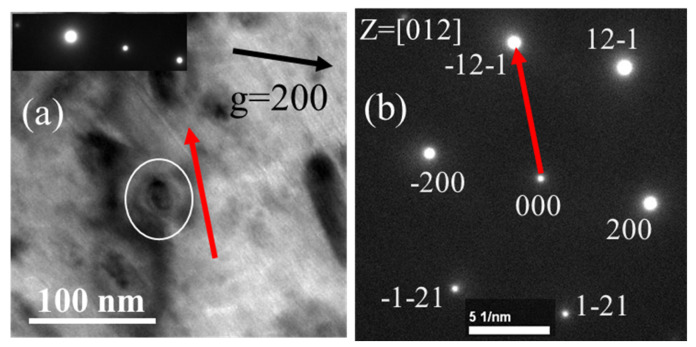
The TEM image of the dislocation loop under [012] zone axis with two-beam diffraction vector of **g** = 200 (**a**), the diffraction pattern of [012] zone axis and 1–21 vectors in [012] zone axis (**b**).

To confirm the above result, the sample is further tilted to the third zone axis [012]. Figure 7 shows that the intersection line between the habit plane (−101) and the projected plane (012) is theoretically alongxt [1–21] direction. Figure 8 experimentally confirms that the longest axis direction of the dislocation loop under the [012] zone axis is along the [1–21] direction.

#### 3.2.4. Analyzing the Nature of the Dislocation Loop

At present, the Burgers vector of the analyzed dislocation loop has been identified as [100] under the [001] zone axis and further confirmed by the inside–outside contrast under the [113] zone axis. Moreover, based on the geometry relationships between the analyzed dislocation loop and the projected planes under the [001] and [113] zone axes, the normal direction of the habit plane has been determined to be ***n*** = [−101], which is further confirmed under the [012] zone axis. Since ***b***·***n*** < 0, the dislocation loop is identified as interstitial type.

## 4. Discussion

The inside–outside method relies on the “inside” or “outside” contrast of a loop in the images under plus or minus diffraction vectors ***g***, separately. With the FS/RH convention, the deviation parameter $s_{g}>0$, and the corrected coordinate in Figure 4, the dislocation loops exhibit outside contrast when ***g***·***b*** < 0, while they show inside contrast when ***g***·***b*** > 0 [28]. The inside–outside technique is straightforward when applied to pure edge dislocation loops, as the habit plane of a pure edge loop is perpendicular to the Burgers vector ***b***, allowing the habit plane to be readily determined once the Burgers vector is known. The procedure is summarized as follows. The first step is correctly indexing the diffraction patterns, where attention needs to be paid to whether an inverse relation exists between the images and the diffraction patterns. Second is determining the Burgers vector with a plus or minus sign, i.e., ±***b***. In principle, it needs a minimum of two different g vectors of non-colinear vectors that make ***g***·***b*** = 0 and some observable g conditions to obtain. In practice, it can be simplified if there are only limited possible Burgers vector types in one specified material. One example is the dislocation loop in Figure 5, and the corresponding examination can be seen in Section 3.2.2. Third, the sign of the loop Burgers vector can be determined from the inside–outside contrast under a pair of g vectors with ***g***·***b*** ≠ 0. The fourth step is to examine the habit plane of the dislocation loop, being either the same or opposite to *b* and keeping its direction from the sample upwards. Finally, loops with ***b***·***n*** < 0 are identified as interstitial loops, while those with ***b***·***n*** > 0 are determined to be vacancy loops.

However, for non-edge loops, the application of the inside–outside technique is not straightforward. It is still applicable for loops if it is possible to find the upper limit angle $\varphi_{m}$ between ***b*** and ***n***. Then, according to the geometry between the loops and the crystal lattice orientation, a safe zone can be determined to make sure that the loops cannot flip over. Maher and Eyre determined the safe zone as an angle α between ***b*** and the crystal orientation ***z*** being below (90° − $\varphi_{m}$) [29]. An example is the ½<111> loops in bcc structural materials. They are mostly prismatic loops and generally considered to originate from faulted loops in {110} planes; thus, their $\varphi_{m}$ is the angle between <111> and <110>, which is 35°. Therefore, for ½<111> loops, when the angle between ***b*** and ***z*** is smaller than 55°, the loop is in the safe zone [30]. Nevertheless, some research reported other habit planes, such as the {112} plane [26], which is out of discussion in the present method demonstration.

**Figure 9:**
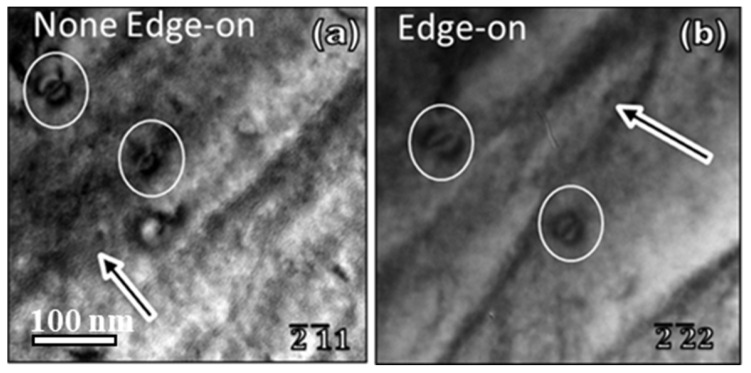
Double-bean contrast of dislocation loops in non-edge-on position and edge-on position [15].

For dislocation loops with totally unknown information, the inside–outside method is difficult to execute. The most challenging thing is to determine the habit plane. In general, people use the edge-on contrast to find the loop normal direction. However, there are several difficulties in using this edge-on contrast approach. Due to the tilting limitation of the sample holder in TEM with α < ±30° and β < ±30° in a typical double-tilt holder, in most cases it is impossible to tilt the loop to an edge-on position. Even though a loop is tilted to an edge-on contrast position, the exact loop normal direction cannot be exactly determined. For example, in Figure 9 two pure edge loops in the (−1–11) plane [15] not only show edge-on contrast under the ***g*** = −2–22 two-beam diffraction condition, where the habit plane seems to be in ±(−1–11), but also show likely edge-on contrast under ***g*** = −2–11, where the habit plane is likely in ±(−2–11) plane. Therefore, only using the edge-on method to determine the habit plane of a dislocation loop can be misleading.

**Figure 10:**
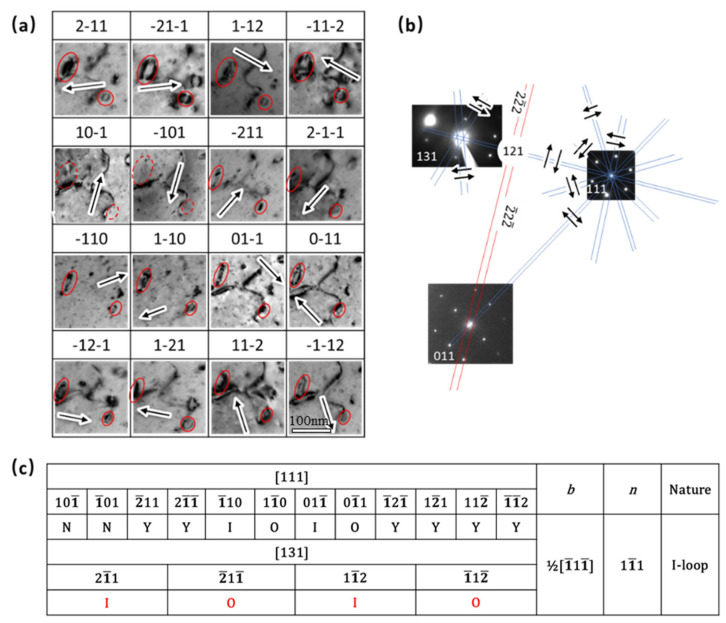
The improved inside–outside method to analyze the habit plane and nature of dislocation loops. (**a**) Images of dislocation loops obtained from different two-beam conditions; (**b**) A schematic shows the two-beam conditions in a Kikuchi map with three zone axes; (**c**) The visibilities and inside outside contrasts of the dislocation loops in (**a**). Note: the arrows represent the g vectors of two-beam conditions; “Y” means visible; “N” means invisible; “O” means visible in outside contrast; “I” means visible in inside contrast; the Burgers vector and nature of the loops were determined based on the data from [111] zone axis.

To address the misleading of the habit plane determination based on the edge-on contrast, a method combining the edge-on contrast and the inside–outside approach has been proposed by us, which is termed the improved inside–outside method [15]. Figure 10 provides an example of this method, where two zone axes are tilted, and several corresponding two-beam images are captured, as shown in Figure 10a. The two beam conditions are indicated by the arrows in Figure 10b. Figure 10c summarizes the visibility and the inside or outside contrast in each two-beam condition. According to our previous studies, the Burgers vector of dislocation loops in ion irradiated pure Cr at 550 °C is ½<111> [3,15]. The two dislocation loops are invisible when ***g*** = ±[10–1], which indicates that their Burgers vector should be ±½[1–11]. Based on the inside–outside contrast (outside contrast ***g***·***b*** < 0, inside contrast ***g***·***b*** > 0) in two-beam condition images on the [111] zone axis, the Burgers vector of the two dislocation loops is determined to be ½<−11–1>. However, when tilting the sample to the [131] zone axis, the inside–outside contrast is inverted, which means that ***g***·***b*** > 0 shows outside contrast and ***g***·***b*** < 0 shows inside contrast. It indicates that the loops have been flipped over when the sample is tilted from [111] to [131]. In addition, when the image is taken under the two-beam condition of ***g*** = [01–1] between the zone axes of [111] and [121], the dislocation loops show edge-on contrast along the ±(1–11) plane. Thus, the ±(1–11) plane should be the habit plane of the loops. Since the normal direction of the habit plane of the loop should be at an acute angle to the electron beam (upwards direction [111]), the habit plane then should be (1–11). Therefore, the two loops with Burgers vectors of ½[−11–1] in the (1–11) plane are determined to be interstitial type (***b***·***n*** < 0).

The stereo-imaging [31,32,33] method is another possible way to determine the habit planes of dislocation loops. It is based on a great reconstruction of 2D images, which are taken in the two-beam condition, to 3D figures, providing critical insights into dislocations’ spatial distribution and crystallographic orientation. According to the crystallographic orientation of dislocation loops with the crystalline materials in the stereo 3D image, the habit plane of the dislocation loop can be obtained. However, the overlap of dislocation loops and the difficulty of precise reconstruction limit its application [31,32,33].

Another method has been proposed for determining the nature of dislocation loops based on their morphology in the face-centered cubic (fcc) material without requiring the habit plane; specifically, circular loops are identified as interstitial type in all fcc materials, whereas segmented loops are vacancy type in high-SFE alloys but exhibit variable characters (mixed or ambiguous type) in low-SFE and high-entropy alloys [34]. However, this approach requires prior classification according to the stacking fault energy (SFE) of the fcc material.

In hcp zirconium alloys, Liu and Han have determined the habit planes of loops based on the projected loop morphology observed in transmission electron microscopy (TEM), assuming that dislocation loops are purely prismatic [35]. This approach relies on the premise that a unique projected shape corresponds exclusively to pure prismatic loops on a given imaging plane; however, if other loop types—such as mixed or non-prismatic loops—can produce similar projected morphologies, the identification of the habit plane may be ambiguous.

The present method demonstrated in the results is a new method to analyze the habit planes of dislocation loops. Compared with the above methods, this method is easy to execute because only two images are required in different zone axes. Through calculation, the habit plane can be obtained precisely. The advantage of this method is that it does not require the dislocation loop to be tilted to an edge-on position in the TEM. The essence of this method lies in the fact that two non-parallel lines define a plane; therefore, it is applicable to determining the habit planes of dislocation loops in materials with any crystallographic structure, of course including fcc and hcp structures.

## 5. Conclusions

In the present study, a new method to determine the habit planes of dislocation loops is proposed and demonstrated in a bcc structural material. This method is readily implementable without requiring an edge-on tilting position in TEM. Additionally, we outline and discuss the practical approaches to determine the nature of dislocation loops as well as their habit planes. The inside–outside method is systematically elucidated, ranging from the most rigorous formulation to simplified variants adaptable to diverse scenarios.

## Data Availability

The original contributions presented in this study are included in the article. Further inquiries can be directed to the corresponding author.
